# Effects of Genetic Risk on Incident Type 2 Diabetes and Glycemia: The T2D-GENE Lifestyle Intervention Trial

**DOI:** 10.1210/clinem/dgae422

**Published:** 2024-06-18

**Authors:** Maria Anneli Lankinen, Petrus Nuotio, Susanna Kauppinen, Noora Koivu, Ulla Tolonen, Katriina Malkki-Keinänen, Anniina Oravilahti, Teemu Kuulasmaa, Matti Uusitupa, Ursula Schwab, Markku Laakso

**Affiliations:** Institute of Public Health and Clinical Nutrition, University of Eastern Finland, 70211 Kuopio, Finland; Institute of Public Health and Clinical Nutrition, University of Eastern Finland, 70211 Kuopio, Finland; Institute of Public Health and Clinical Nutrition, University of Eastern Finland, 70211 Kuopio, Finland; Institute of Public Health and Clinical Nutrition, University of Eastern Finland, 70211 Kuopio, Finland; Institute of Public Health and Clinical Nutrition, University of Eastern Finland, 70211 Kuopio, Finland; Institute of Public Health and Clinical Nutrition, University of Eastern Finland, 70211 Kuopio, Finland; Institute of Clinical Medicine, Internal Medicine, University of Eastern Finland, 70211 Kuopio, Finland; Institute of Clinical Medicine, Internal Medicine, University of Eastern Finland, 70211 Kuopio, Finland; Institute of Public Health and Clinical Nutrition, University of Eastern Finland, 70211 Kuopio, Finland; Institute of Public Health and Clinical Nutrition, University of Eastern Finland, 70211 Kuopio, Finland; Department of Medicine, Endocrinology and Clinical Nutrition, Kuopio University Hospital, Wellbeing Services County of North Savo, 70210 Kuopio, Finland; Institute of Clinical Medicine, Internal Medicine, University of Eastern Finland and Kuopio University Hospital, 70211 Kuopio, Finland

**Keywords:** type 2 diabetes, genetics, diet, clinical trial, human, lifestyle, intervention

## Abstract

**Context:**

Lifestyle intervention prevents or delays type 2 diabetes (T2D) in subjects at a high risk of T2D. However, it is not known whether genetic variants modify the effect on incident T2D during lifestyle intervention.

**Objective:**

To investigate whether a low or high genetic risk has effects on incident T2D in a group-based lifestyle intervention study.

**Methods:**

The T2D-GENE trial involved 973 men from the Metabolic Syndrome in Men (METSIM) cohort, aged 50-75 years, body mass index ≥25 kg/m^2^, fasting plasma glucose 5.6-6.9 mmol/L, hemoglobin A1c < 48 mmol/mol, and either a low or high genetic risk score for T2D. There were 2 intervention groups, a low (n = 315) and high genetic risk for T2D (n = 313). They were provided with a 3-year group-based intervention with access to a web portal focused on healthy diet and physical activity. There were also corresponding population-based control groups at low (n = 196) and high (n = 149) genetic risk for T2D who had two laboratory visits (0 and 3 years) and general health advice as a part of their METSIM cohort protocol. The primary outcome was incident T2D, and a secondary outcome was glycemia.

**Results:**

The intervention significantly lowered the risk of T2D among the participants with a high genetic risk for T2D [hazards ratio (HR) 0.30, 95% confidence interval (CI) 0.16-0.56, *P* < .001) whereas in the low genetic risk group the effect was not significant (HR 0.69, 95% CI 0.36-1.32, *P* = .262). The intervention effect was not significantly different between the high and low genetic risk groups (*P* = .135). The intervention significantly ameliorated the worsening of glycemia and decreased weight both in the low and high genetic risk groups.

**Conclusion:**

Our results showed that individuals with a high genetic risk for T2D benefitted from a low-cost group-based intervention focusing on healthy diet and physical activity. Therefore, all individuals at risk of T2D should be encouraged to make lifestyle changes regardless of genetic risk.

The prevalence of diabetes mellitus will likely increase globally from 537 million adults (20-79 years) to 643 million by 2030 and 783 million by 2045, according to the International Diabetes Federation (1). Diabetes is and remains a serious and growing challenge to public health given the risk of developing several micro- and macrovascular complications.

Both genetic and environmental/lifestyle factors contribute to the risk of type 2 diabetes (T2D). Genome-wide association studies have identified >500 common variants increasing the risk of T2D (2, 3) Several previous studies have demonstrated that lifestyle intervention is effective in the prevention of T2D (4-9). The Finnish Diabetes Prevention Study (DPS) and Diabetes Prevention Program (5) showed that lifestyle intervention can lower the risk of T2D by 58% compared to the control group. A recent systematic review and meta-analysis of 7 randomized controlled T2D prevention trials reported that the lifestyle changes lowered the risk of incident T2D by 53% (10). These intervention studies included participants with impaired glucose tolerance (IGT), and their sample size was <600 individuals, except for the Diabetes Prevention Program including 3234 participants and a mean age ranged from 44 to 55 years. All these trials applied an individual-based intervention. A Japanese trial including 641 participants with either isolated impaired fasting glucose (IFG) or IFG + IGT demonstrated more effective T2D prevention in the participants with both IFG and IGT compared to isolated IFG (11).

Previously published lifestyle intervention studies have investigated the significance of genetic factors on the risk of incident T2D based only on single risk variants or a narrow risk score (12-14). We previously showed that a genetic risk score (GRS) including 76 genetic risk variants significantly increased the risk of incident T2D (15). The aim of the current study was to investigate whether the effect of lifestyle intervention [healthy diet and physical activity (PA)] on the prevention of T2D differs between individuals with a high or low genetic risk for T2D by applying a group- and internet-based approach to lifestyle changes.

## Methods

### Study Design

This T2D-GENE trial (ClinicalTrials.gov ID: NCT02709057) was performed at 1 site at the University of Eastern Finland, Kuopio, Finland. The study protocol including the calculation of the sample size has been previously described in detail (16). This trial was accepted by the Ethics Committee of the Hospital District of Northern Savo on February 9, 2016 (no. 71/2016) and was performed in accordance with the International Declaration of Helsinki 2013 (17). Written informed consent was obtained from each participant included in the study. The T2D-GENE trial started on April 4, 2016, and the last 3-year visit was on October 19, 2021. The participants were recruited from the Metabolic Syndrome in Men (METSIM) study including 10 197 Finnish men, aged from 45 to 73 years at baseline (18, 19).

### Screening and Inclusion Criteria

Eligible participants of the T2D-GENE trial were individuals belonging to the METSIM cohort (18, 19) having IFG (fasting plasma glucose 5.6-6.9 mmol/L), with IGT (2-hour glucose 7.8-11.0 mmol/L) or without IGT (2-hour glucose <7.8 mmol/L), hemoglobin A1c (HbA1c) < 48 mmol/mol (<6.5%), age 50-75 years, body mass index (BMI) ≥25.0 kg/m^2^, and a low or high GRS (≤76 or ≥80) [Supplementary Table S1 (20)]. The nonweighted GRS for T2D (15) was calculated as a sum of 76 genetic variants known to increase the risk of T2D in 2016 (21, 22). The baseline measurements were performed in their METSIM follow-up visit. A total of 83.6% had isolated IFG and 16.4% had both IFG and IGT.

### Intervention and Control Groups

**Figure 1.**
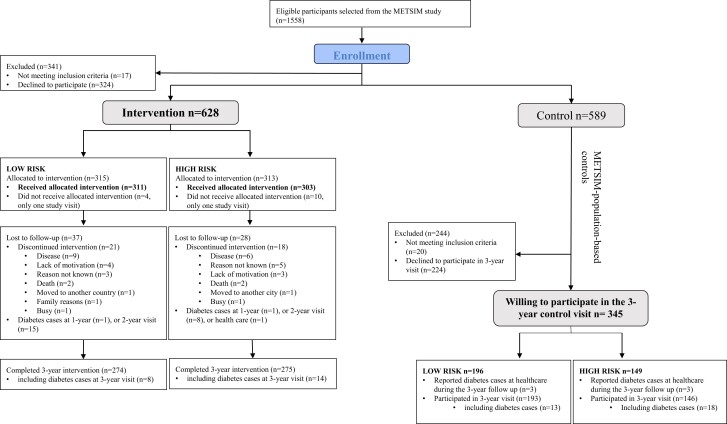
Flow chart of the T2D-GENE study.

This study consisted of an intervention arm with 2 groups (low and high genetic risk groups) and a corresponding population control arm with 2 groups (low and high genetic risk groups) (Fig. 1). A total of 628 participants fulfilling the inclusion criteria were recruited in the T2D-GENE study intervention arm. According to the original study design, 313 participants in the intervention arm had a high genetic risk (GRS 83.3 ± 3, mean ± SD) and 315 participants had a low genetic risk for T2D (GRS 72.4 ± 3.1). Fourteen participants discontinued the intervention after the first group meeting and were excluded from statistical analyses. The dropouts (6.4%) were otherwise similar as completers but had a higher BMI (29.1 vs 27.8 kg/m^2^), waist circumference (105 vs 102 cm), and serum triglycerides (1.32 vs 1.23 mmol/L).

Population controls (n = 589) were selected from the METSIM cohort having similar inclusion criteria as the participants in the intervention group, but they were invited only for 3-year measurements. Baseline measurements were conducted as a part of the METSIM cohort protocol, similarly as in the intervention arm. A total of 345 of the invited participants were willing to attend the 3-year laboratory visit. Control participants were unaware that they were part of the T2D-GENE-study during the 3-year follow-up, and they received general health advice as a part of the METSIM cohort protocol at the baseline visit. In the control arm, 149 participants had a high genetic risk (GRS 83.2 ± 2.9) and 196 a low genetic risk (GRS 72.5 ± 2.8) for T2D.

The trial participants, laboratory technicians, and clinical nutritionists were blinded to the genetic risk of the participants.

### Lifestyle Intervention

The 3-year intervention program has been previously published (16, 23). The intervention included group sessions on the importance of a healthy diet and PA. In the intervention groups, dietary intake was measured by 4-day food records at baseline and 4 times during the intervention. The participants were given individual written feedback on food records by clinical nutritionists to support participants’ adherence to the health-promoting diet.

Dietary guidance in the intervention groups followed Nordic (24) and Finnish nutrition recommendations, emphasizing appropriate energy intake; meal frequency; consumption of fruits, vegetables, and berries; and quality of dietary fat and carbohydrates, including fiber and sugar intake. Regarding weight management, the minimum goal for the participants was weight maintenance. The goal for PA was brisk walking or other types of PA ≥30 minutes/day at least 5 times a week. We encouraged participants to self-report their weekly PA through the web portal or in paper form for motivation. We evaluated leisure time PA using a questionnaire that measured the frequency and duration of PA on a 4-point scale.

The first year of the intervention included 3 group meetings for all participants and 2 additional group meetings for participants having BMI >28 kg/m^2^ at baseline. Other visits to our research facility were at 1, 2, and 3 years, including laboratory measurements. The web portal was also used to distribute material on healthy food choices and PA and to motivate virtual discussion between the participants and clinical nutritionists.

### Outcomes

The primary outcome of the T2D-GENE trial was incident diabetes defined by fasting plasma glucose ≥7.0 mmol/L or 2-hour glucose in an oral glucose tolerance test ≥11.1 mmol/L or HbA1c ≥ 48 mmol/mol (≥6.5%) or drug treatment for diabetes during the 3-year intervention (18). We identified incident diabetes in the intervention arm by measuring HbA1c at 1 year and using HbA1c and oral glucose tolerance tests at 2 and 3 years. The participants of the control arm had visits at 0 and 3 years, and their glucose tolerance status was measured by HbA1c and an oral glucose tolerance test.

Secondary outcomes were 3-year changes in (1) glucose area under the curve (AUC) in an oral glucose tolerance test (glucose measured at 0, 30, and 120 minutes), (2) insulin secretion (Disposition Index), (3) insulin sensitivity (Matsuda insulin sensitivity index), as previously described (18, 25).

### Clinical and Laboratory Measurements

Fat mass was measured by bioimpedance (BIA 101, Akern SRL, Italy). Waist circumference was measured at the midpoint between the lateral iliac crest and the lowest rib. Laboratory analyses were performed after 12 hours of fasting. We measured HbA1c and performed an oral glucose tolerance test (75 g of glucose) to evaluate glucose tolerance according to the American Diabetes Association criteria (26). We measured plasma glucose by enzymatic hexokinase photometric assay (Konelab Systems Reagents, Thermo Fisher Scientific, Vantaa, Finland), HbA1c by Tosoh G7 glycohemoglobin analyzer (Tosoh Bioscience, Inc. San Francisco, CA, USA), insulin by immunoassay (Liaison Insulin, DiaSorin S.p.A, Saluggia, Italy, RRID:AB_3099584), and total cholesterol and low-density lipoprotein cholesterol by enzymatic colorimetric tests (Konelab Systems Reagents; Thermo Fisher Scientific, Vantaa, Finland).

### Genotyping

Genotyping was performed using either HumanOmniExpress BeadChip-12v1 (Illumina, San Diego, CA, USA; 733 202 markers) or HumanExome-12v1.1 Beadchip (Illumina, 247 870 markers) as previously described (15).

### Statistical Analyses

The sample size was calculated based on the Finnish DPS study, where the incidence of T2D was 3%/year in the intervention group and 6%/year in the control group (5). Assuming similar rates of incident T2D, only 60 individuals/group would have been needed to demonstrate statistically significant difference between the intervention and control groups (α = .05, β = .95). We anticipated that our group-based intervention might be less efficient than the individual-based approach in the DPS study. With half of the effect observed in the DPS study, 267 participants would have been needed. Assuming a 10% dropout rate in this trial, a sample size of 300/each group provides 95% power to demonstrate a statistically significant reduction in incident T2D in the intervention groups compared with the control groups.

Statistical analyses were conducted using R (v4.2.2; R Core Team 2022). Descriptive statistics are presented as mean (±SD), number (%), or median (interquartile range) for variables with absolute skewness >1. In the figures, values are shown as mean [95% confidence interval (CI)]. Differences at baseline were tested by independent samples *t*-test. Chi-squared tests were used for categorical variables. Cumulative incidence of T2D was estimated using Kaplan–Meier event curves and between-group differences using Cox proportional hazard regression models (R survival package, v3.4.0). Intervention and control arms were compared in all participants and by stratifying the analysis by GRS group. In both cases, the treatment arm was included as the independent variable. Intervention effect was then compared between the low and high GRS groups by including a treatment arm × GRS group interaction term in the model. Schoenfeld residuals indicated no significant violation of the proportional hazard assumption. Multiple comparison correction was addressed with Benjamini–Hochberg false discovery rate correction (27).

Changes in laboratory measurements and dietary intake were tested using repeated measures linear mixed models (R lme4 package v1.1–31). Models were fit using the restricted maximum likelihood method while ignoring missing observations. Skewed variables were log10-transformed prior to statistical analyses. For laboratory measurements, analyses were run individually for the low and high GRS groups, including the outcome of interest as a dependent variable, subject identifier as a random effect (random intercept) and time point (baseline or year 3), treatment arm (intervention or control), and time point × treatment arm interaction as covariates. To compare the effects of the intervention between low and high GRS groups, we ran similar models pooling the data from the low and high GRS groups and added GRS group into the model as time point × treatment arm × GRS group interaction. For dietary changes within the intervention arm, models included time point, GRS group, and time point × GRS group interaction as covariates.

## Results

Baseline characteristics of the participants are given in Table 1. In the low genetic risk groups, participants belonging to the intervention group were older than the participants in the population control group (false discovery rate *P* = .008).

**Table 1.** Baseline characteristics of the study participants in the 4 study groups

	Low genetic risk				High genetic risk			
Variable	Intervention (n = 311)	Control (n = 196)	*P^a^*	FDR *P^a^*	Intervention (n = 303)	Control (n = 149)	*P^b^*	FDR *P^b^*
Age, mean (SD), years	65.6 (5.7)	63.8 (5.7)	<.001	.008	64.8 (6.1)	63.8 (5.4)	.078	.472
Weight, median (IQR), kg	89.0 (82.0;96.5)	88.0 (81.0;96.1)	.244	.488	87.0 (81.4;93.3)	86.0 (81.0; 94.0)	.807	.857
Body mass index, median (IQR), kg/m^2^	28.2 (26.6;30.6)	27.8 (26.2;29.9)	.123	.310	27.7 (26.5;29.5)	27.5 (26.2; 29.7)	.682	.857
Waist circumference, median (IQR), cm	103.0 (98.0;109.5)	101.0 (97.0;107.0)	.046	.275	101.0 (97.0;106.0)	101.0 (97.0;106.0)	.840	.857
Fat mass, mean (SD), %	27.4 (6.0)	26.8 (5.9)	.353	.605	26.3 (5.5)	25.9 (5.6)	.464	.857
Fasting plasma glucose, mean (SD), mmol/L	6.0 (0.3)	6.1 (0.4)	.099	.310	6.0 (0.3)	6.1 (0.3)	.037	.449
120 minutes plasma glucose, mean (SD), mmol/L	6.3 (1.6)	6.2 (1.8)	.417	.626	6.2 (1.6)	6.4 (1.6)	.155	.620
HbA1c, mean (SD), mmol/mol	37.5 (3.2)	37.7 (3.1)	.569	.759	37.4 (3.1)	37.5 (3.3)	.857	.857
Total cholesterol, mean (SD), mmol/L	5.0 (1.0)	5.0 (1.0)	.998	.998	5.1 (1.0)	5.1 (1.1)	.812	.857
LDL cholesterol, mean (SD), mmol/L	3.1 (0.9)	3.1 (0.9)	.908	.991	3.1 (0.9)	3.1 (1.0)	.854	.857
HDL cholesterol, mean (SD), mmol/L	1.4 (0.4)	1.4 (0.4)	.644	.773	1.4 (0.4)	1.4 (0.4)	.249	.747
Triglycerides, median (IQR), mmol/L	1.2 (0.9;1.6)	1.2 (0.9;1.6)	.129	.310	1.2 (0.9;1.6)	1.2 (0.9; 1.6)	.330	.792

Abbreviations: FDR, false discovery rate; HbA1c, glycosylated hemoglobin; HDL, high-density lipoprotein; IQR, interquartile range; LDL, low-density lipoprotein.

^*a*^Baseline differences between the intervention and control arms (independent samples t-test) in the participants with a low genetic risk for type 2 diabetes.

^*b*^Baseline differences between the intervention and control arms (independent samples *t*-test) in the participants with a high genetic risk for type 2 diabetes.

**Figure 2.**
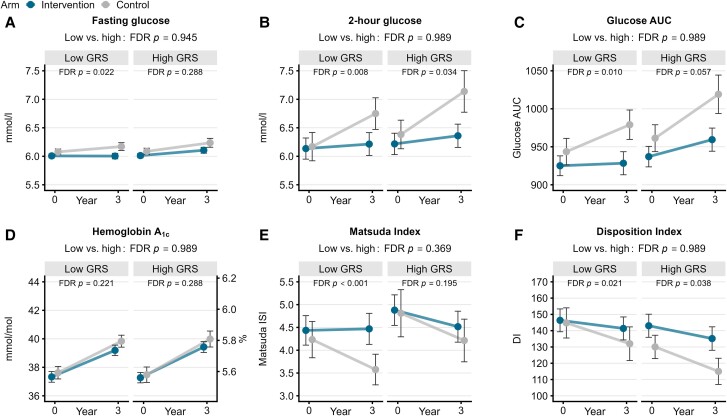
Changes (mean, 95% confidence interval) in fasting glucose (A), 2-hour glucose (B), glucose area under the curve (C), hemoglobin A1c (D), Matsuda insulin sensitivity index, (E), and disposition index (F) at baseline and the 3-year visits in the intervention and the control groups among the participants with low and high genetic risk for type 2 diabetes. False discovery rate *P*-values are for intervention effect (Time × Arm) within the genetic risk group. Abbreviation: GRS, genetic risk score.

The primary outcome of the T2D-GENE trial was incident T2D. In the intervention arm, the conversion to diabetes was 7.7% in the low and 7.9% in high genetic risk groups. In the population control arm, 8.2% of the participants in the low genetic risk group and 14.1% of the participants in the high genetic risk group developed T2D. Overall, the intervention lowered the risk of T2D by 52% [hazards ratio (HR) 0.48, 95% CI 0.31-0.75, *P* = .001]. Among the participants with a high genetic risk for T2D, the intervention lowered the risk of T2D by 70% (HR 0.30, 95% CI 0.16-0.56, *P* < .001) whereas among the participants with a low genetic risk for T2D, there was no significant difference in incident T2D between the intervention and control arms (HR 0.69, 95% CI 0.36-1.32, *P* = .262) (Table 2). The intervention was equally effective against worsening of 2-hour glucose and disposition index in both high and low genetic risk groups (Table 3, Fig. 2). Additionally, in the low genetic risk group, the intervention prevented an increase in fasting glucose concentration and glucose AUC and a decrease in the Matsuda index. HbA1c increased similarly in both genetic risk groups and treatment arms.

**Table 2.** Cumulative incidence of T2D in the intervention and control arms stratified by genetic risk for T2D

	Intervention (%)	Control (%)	HR (95% CI) *P*-value*^a^*
Low genetic risk	7.7	8.2	0.69 (0.36-1.32) *P* = .262
High genetic risk	7.9	14.1	0.30 (0.16-0.56) *P* < .001
*P^b^*			.135

Abbreviations: CI, confidence interval; HR, hazards ratio; T2D, type 2 diabetes.

^*a*^Cox regression model.

^*b*^Intervention effect in low vs high genetic risk groups.

**Table 3.** Changes in anthropometric and laboratory measurements from baseline to the 3-year follow-up visits in participants with low and high genetic risk for type 2 diabetes in the intervention and control arms

	Low genetic risk				High genetic risk					
Variable, mean (SD)	Intervention (n = 274)	Control (n = 193)	*P^a^*	FDR *P^a^*	Intervention (n = 275)	Control (n = 146)	*P^a^*	FDR *P^a^*	*P^b^*	FDR *P^b^*
Weight, kg	−1.49 (4.55)	−0.30 (4.29)	.004	.010	−1.69 (4.55)	−0.20 (4.44)	.001	.014	.574	.945
Body mass index, kg/m^2^	−0.31 (1.44)	+0.06 (1.39)	.006	.010	−0.37 (1.46)	+0.08 (1.44)	.002	.014	.618	.945
Waist circumference, cm	−1.25 (5.10)	−0.07 (4.88)	.018	.022	−1.27 (5.03)	−0.49 (5.30)	.145	.248	.630	.945
Fat mass, %	+3.19 (5.01)	+2.64 (4.75)	.238	.238	+3.13 (4.80)	+3.83 (4.99)	.220	.288	.089	.369
Total cholesterol, mmol/L	−0.40 (0.79)	−0.16 (0.77)	<.001	.004	−0.29 (0.77)	−0.23 (0.93)	.316	.345	.123	.369
LDL cholesterol, mmol/L	−0.33 (0.74)	−0.12 (0.69)	<.001	.004	−0.25 (0.67)	−0.27 (0.86)	.769	.769	.015	.183
Fasting plasma glucose, mmol/L	−0.00 (0.41)	+0.09 (0.44)	.019	.022	+0.09 (0.41)	+0.15 (0.44)	.224	.288	.473	.945
120 minutes plasma glucose, mmol/L	+0.09 (1.59)	+0.58 (1.81)	.003	.008	+0.14 (1.74)	+0.75 (2.04)	.009	.034	.967	.989
HbA1c, mmol/mol	+1.85 (2.89)	+2.21 (2.63)	.202	.221	+2.15 (2.88)	+2.51 (2.70)	.240	.288	.989	.989
Glucose, AUC	+4.48 (115.45)	+36.02 (129.04)	.005	.010	+22.65 (122.73)	+57.78 (144.31)	.024	.057	.869	.989
Matsuda ISI	+0.04 (2.42)	−0.70 (2.04)	<.001	<.001	−0.36 (2.52)	−0.62 (2.25)	.097	.195	.120	.369
Disposition index	−5.01 (52.66)	−13.56 (49.17)	.014	.021	−7.94 (53.96)	−15.35 (40.56)	.013	.038	.812	.989

Abbreviations: AUC, area under the curve; HbA1c, glycosylated hemoglobin; FDR, false discovery rate; ISI, insulin sensitivity index; LDL, low-density lipoprotein.

^*a*^Intervention effect (Time × Arm) within the genetic risk group.

^*b*^The difference in intervention effect in the low vs high genetic risk groups (time × arm × genetic risk group).

The participants in the intervention arm lost more weight and had a larger decrease in BMI compared with the population controls, irrespective of genetic risk [Table 3, Supplementary Fig. S1 (20)]. Overall, the effects of the lifestyle intervention were similar in the low genetic risk and high genetic risk groups with respect to anthropometric measurements.

**Figure 3.**
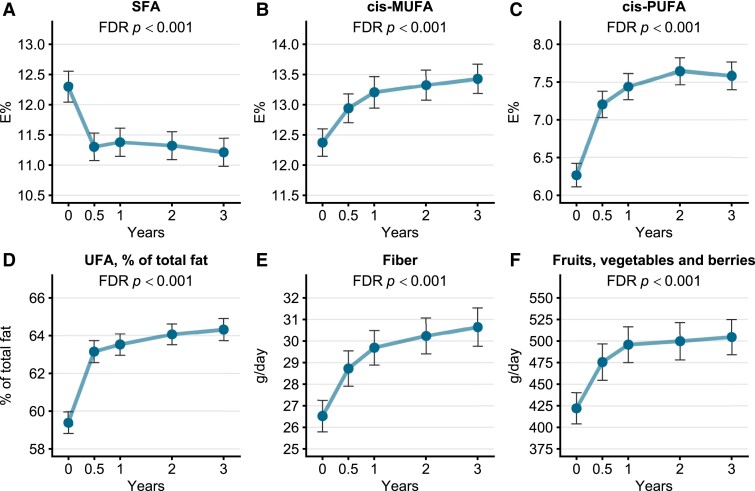
Changes (mean, 95% confidence interval) in the intake of key nutrients (A-E) and fruits, vegetables, and berries (F) during the 3-year intervention in the intervention arm. The low and high genetic risk groups are presented together as there were no significant differences between the genotype groups. Abbreviations: E%, percentage of total energy intake; MUFA, monounsaturated fatty acids; PUFA, polyunsaturated fatty acids; SFA, saturated fatty acids; UFA, unsaturated fatty acids.

Dietary intake of saturated fatty acids decreased and intakes of monounsaturated fatty acids, polyunsaturated fatty acids, fiber, and fruits, vegetables, and berries increased significantly in the intervention arm during the intervention, similarly in both genotype groups [*P* < .001, Fig. 3, Supplementary Table S2 (20)]. A total of 20% of the participants in the intervention and population control groups increased their PA during the 3-year study. In the intervention group, the participants with high genetic risk were more active compared with the participants with low genetic risk at baseline (*P* = .010), but there were no differences in PA at the end of the 3-year intervention between the high and low genetic risk groups (*P* = .993) [Supplementary Table S3 (20)].

## Discussion

The novel finding of our study was that the lifestyle intervention (healthy diet, PA) significantly prevented the conversion to T2D in the participants with a high genetic risk for diabetes compared with the population controls, whereas no significant difference between the intervention and control arms was observed in the participants with a low genetic risk. However, the intervention effect was not significantly different between the high and low genetic risk groups (*P* = .135), suggesting that the participants having a low genetic risk for T2D also benefitted from lifestyle intervention. The intervention significantly ameliorated worsening of 2-hour glucose, glucose AUC, and disposition index both in the low and high genetic risk groups. In the low genetic risk group, the intervention also prevented an increase in fasting glucose and a decrease in the Matsuda index. These findings suggest that participants in both groups benefitted from lifestyle intervention irrespective of the genetic risk.

In the intervention arm, the conversion to diabetes was 7.7% in the low and 7.9% in the high genetic risk groups. In the population control groups, 8.2% of the participants in the low genetic risk group and 14.1% of the participants in the high genetic risk group developed T2D. Our results are in agreement with the results of 2 previously published T2D prevention trials showing that lifestyle intervention was particularly effective among participants carrying the *TCF7L2* risk variant (12, 13).

The participants in the population control arm had only 2 visits (0 and 3 years), and they were not exposed to the intervention protocol. Incident T2D in the population control arm is probably underestimated because an oral glucose tolerance test was performed only twice (0 and 3 years) compared with 3 times in the intervention groups. Overall, incident T2D was quite low in all study groups (7.7%-14.1%) compared to the Finnish DPS study, where the cumulative incident T2D after four years was 11% in the intervention group and 23% in the control group (5).

We found that glycemia tended to worsen over time in both intervention and control arms. The adverse changes were significantly smaller in the intervention groups compared with the control groups, suggesting that the intervention ameliorated the worsening of glycemia. The intervention significantly lowered BMI and waist circumference in both genetic risk groups. Fat mass increased both in the intervention and control groups, which is probably attributable to aging that is associated with a decrease in muscle mass and an increase in fat mass (28).

We used IFG as an inclusion criterion in contrast to the majority of previous T2D prevention trials, which used IGT (5-9), apart from 3 Asian trials (11, 29, 30). IFG and IGT have different pathophysiology given the fact that genetic variants for fasting and 2-hour glucose are only partially identical (31). Previously published T2D prevention trials included participants whose mean age was ≤56 years (5-9). Especially in elderly populations, T2D and obesity are reaching epidemic proportions, but clinical trial data on strategies to prevent T2D in the elderly are largely missing. Our trial is the first to show that a lifestyle intervention is successful also in participants with a mean age ≥60 years.

Our T2D prevention program utilized a group-based approach instead of an individual-based intervention. Our results suggest that a group-based approach is efficient in the prevention of T2D, as reported in a previous study (32). We used a web portal that enabled the distribution of material and virtual discussion between participants and clinical nutritionists.

Our study has important clinical implications. We found that lifestyle intervention is effective for T2D prevention in individuals at high genetic risk. Lifestyle intervention is also beneficial in individuals with a low genetic risk when considering its effect on glycemia. This suggests that a similar lifestyle intervention could be provided for all individuals at risk of T2D, independent of the genetic risk, and individualized intervention programs are not justified based on these results. However, T2D is a heterogenous disease, and therefore a precise nutrition approach might be beneficial for some subgroups of T2D. Our group-based intervention program is substantially less costly compared to individual counseling, and it is applicable to primary care.

Limitations of our study are that our study included only men and we were not able to recruit 300 participants in the population control groups. We calculated that at least 189 individuals are needed in the low genetic risk groups to obtain significant results with α = .05, β = .95 and the HR of 0.69. We had 196 participants in the low genetic risk control group showing that our study was not underpowered to obtain statistical significance also in the low genetic risk groups. Another limitation was that we did not have a randomized control arm. Instead, we had a population control arm with the same inclusion criteria and duration of the follow-up as the intervention participants. As mentioned earlier, incident T2D in the population control arm was probably underestimated because they had measurements only at baseline and year 3. Despite this limitation, the intervention lowered the risk of T2D by 52% compared with the population control.

## Conclusions

Our T2D-GENE trial shows that T2D can be prevented or delayed by a low-cost group-based approach focusing on healthy diet and physical activity in middle-aged and elderly men, especially in the participants with a high genetic risk for T2D. Lifestyle intervention was equally effective in lowering glycemia and weight compared to the population control groups in the participants with either high or low genetic risk for T2D. Therefore, our results suggest that all individuals at risk of T2D should be encouraged to make lifestyle changes regardless of genetic risk.

## Data Availability

Restrictions apply to the availability of data generated or analyzed during this study to preserve the confidentiality of the participants. The corresponding author will, on request, detail the restrictions and any conditions under which access to some data may be provided.

## Clinical Trial Information

ClinicalTrials.gov ID: NCT02709057.
